# Psychological status and related factors of resident physicians during the release of COVID-19 pandemic restrictions in China

**DOI:** 10.3389/fpubh.2024.1322742

**Published:** 2024-04-17

**Authors:** Qing Zhang, Ruibo Pan, Qi Pan, Yandan Qian, Xiao Zhou, Qiaozhen Chen

**Affiliations:** ^1^Department of Psychiatry, The Second Affiliated Hospital, Zhejiang University School of Medicine, Hangzhou, China; ^2^Department of Psychiatry, The Third People's Hospital of Jiashan County, Jiaxing, China; ^3^Department of Psychology and Behavioral Science, Zhejiang University, Hangzhou, China; ^4^Key Laboratory of Medical Molecular Imaging of Zhejiang Province, Hangzhou, China

**Keywords:** COVID-19, anxiety, depression, somatic symptoms, job burnout, vicarious trauma, resident physicians

## Abstract

**Background:**

Resident physicians at the standardized training stage had undergone significant physical and mental stress during the release of the COVID-19 pandemic restrictions at the end of 2022 in China. This study aimed to investigate the psychological status (including anxiety, depression, somatic symptoms, job burnout, and vicarious trauma) of resident physicians and identify its influencing factors under these special periods.

**Methods:**

Survey was conducted one month after the release of the COVID-19 pandemic restrictions on resident training physicians from a tertiary first-class hospital in Zhejiang, China. Resident physicians completed the psychological status questionnaire. Chi-square tests, Mann–Whitney U tests, and logistic regression analyses were used to estimate the group differences and variable associations.

**Results:**

The prevalence of anxiety, depression, and somatic discomfort in this study was 20.88, 28.53, and 41.47%, respectively. Female resident physicians were more likely to experience somatic symptoms [adjusted odds ratio (*OR*) = 2.36, 95% confidence interval (*CI*): 1.33–4.18]. Resident physicians with problem-focused coping styles were less prone to psychological health issues [depression (adjusted *OR* = 0.92, 95% *CI*: 0.88–0.96), anxiety (adjusted *OR* = 0.94, 95% *CI*: 0.90–0.98), somatic symptoms (adjusted *OR* = 0.93, 95% *CI*: 0.89–0.97), job burnout (adjusted *OR* = 0.91, 95% *CI*: 0.87–0.96) and vicarious trauma (adjusted *OR* = 0.94, 95% *CI*: 0.90–0.98)]. Inversely, resident physicians with emotion-focused coping styles and experienced negative life events were more prone to psychological health issues.

**Conclusion:**

Resident training physicians had a high risk of anxiety, depression, and somatic symptoms under the special COVID-19 pandemic restriction release period. Females, with lower training stages, degrees, negative life events, and emotion-focused coping styles had a disadvantaged effect on psychological status. The medical teaching management department needs to monitor and reduce the workload and working hours of resident physicians, ensure sufficient sleep time, and pay attention to the psychological status of resident physicians. By strengthening regular communication and mental health education or intervention, which can help them improve their ability to cope with complex tasks.

## Introduction

In the past three COVID-19 pandemic years, medical workers have endured extreme psychological pressure. A series of studies have been conducted about the psychological health status of medical workers (1, 2), including medical students (3, 4). During the study period, medical workers were actively involved in the care of COVID-19 patients (1, 2). Both were found a high prevalence of anxiety and depression, but there were few studies on resident training physicians. The resident physicians refer to medical graduates who make medical practices under the direct or indirect supervision of a senior clinician for three years in a qualified hospital training base. As is well known, outbreaks of infectious diseases have psychological influences on medical workers and the general population (1). Medical workers often encounter various mental problems under high pressure and high-risk epidemic prevention situations (5). At the beginning of the outbreak of COVID-19, there has a study found that one-sixth of people reported moderate to severe depressive symptoms and more than a quarter of people reported moderate to severe anxiety symptoms (6); Further longitudinal study found that there was no remarkable difference in anxiety and depressive levels (7).

Standardized training for resident physicians is the most critical stage from medical students to independent doctors (8, 9), residents would face multiple pressures not only in daily life and work but also in various training and assessments. Some physicians are prone to job burnout, which is a mental syndrome caused by persistent work pressure leading to emotional exhaustion, personality disintegration, and impaired personal achievement (10). The topic of burnout has dominated research on medical personnel (11). Previous studies have shown that the prevalence of burnout among physicians was particularly high. The prevalence of job burnout among medical workers (12), primary care practitioners (13), practicing physicians (14), and medical students (15) reached 24, 40.3, 50, and 71%, respectively. Medical workers experienced unprecedented stress related to work since the outbreak of COVID-19 (16). At the end of 2022, China released the COVID-19 epidemic restriction, and a widespread pandemic trend emerged. Resident physicians assumed important responsibilities for the treatment of COVID-19 patients. Due to the lack of human resources, the workload was increased significantly, for instance, during the outbreak of the epidemic, many frontline medical staff worked an average of 16 h or more per day (17). Some resident physicians persisted in work and practice tasks despite their own infections.

In this situation, the likelihood of psychological problems among medical workers increased. As mentioned earlier, there have been studies of anxiety, depression, and job burnout among medical workers, but there was relatively little discussion on vicarious trauma for resident physicians during the epidemic. In addition to common psychological problems such as anxiety and depression, vicarious trauma was also within the scope of our research. Previous studies on major traumatic events (such as post-disaster studies) usually involved the assessment of vicarious trauma (18). When medical workers took care of patients infected with COVID-19, the risk of infection increased sharply. They had empathy for patients’ uncomfortable symptoms and pain, and were prone to vicarious trauma symptoms, further leading to serious physical and mental distress (19, 20).

The outbreak of infectious diseases meant that resident physicians were facing many stressful events. The epidemic itself was a negative life event for everyone, and its impact on medical workers was even greater (16). The consequences faced on the pandemic were all negative events such as inconvenient living, increased work pressure, and shortage of medical resources (21). A longitudinal study of medical workers found that outcomes such as depression, hopelessness, fatigue, fear of going to work, fear of missing work, and worry about caring for family members that worsened at the start of the pandemic did not statistically improve over time (22). Different individuals may have different outcomes when dealing with negative events in different coping ways. Coping is defined as the cognitive and behavioral strategy to deal with the internal and external needs of an interaction with the environment when he or she judges that such interaction may be trouble to him/herself, even beyond the self-resources (23). Coping includes five conceptual domains: social support, avoidance strategies, a positive attitude, problem orientation and transcendent orientation (24). A study revealed that avoidance, problem orientation, and social support coping worsened professional quality of life, whereas a positive attitude improved it (24). A review of the psychological health condition of medical personnel during COVID-19 pointed out that many situations were normal reactions to the pandemic. They also had negative psychological effects such as burnout, compassion fatigue, anxiety, and depression (25).

During this special period, resident physicians’ psychological health status deserved attention. Most of the psychological health data of medical staff related to the COVID-19 epidemic were during the early stages of the pandemic outbreak. To the best of our knowledge, not much research has been done on mental health in China since the COVID 19 restrictions were lifted. Therefore, our study investigated the prevalence of anxiety, depression, and somatic symptoms among resident physicians around one month after the COVID-19 pandemic was lifted from lockdown, and the relationship between anxiety, depression, somatic symptoms, job burnout, vicarious trauma, and different backgrounds, negative events, social support, coping styles which were factors that might be related to the occurrence of the psychological health issues. The study investigated protective and risk factors for psychological status following China’s lifting of lockdown due to the COVID-19 pandemic. Targeted interventions to enhance the well-being of residents can be guided by identifying populations at higher risk for anxiety, depression, burnout, and secondary traumatic stress.

## Methods

### Participants and procedures

A cross-sectional survey using designed questionnaires including Generalized Anxiety Disorder 7-item (GAD-7), Patient Health Questionnaire 9-item (PHQ-9), Patient Health Questionnaire 15-item (PHQ-15), Maslach Burnout Inventory-General Survey (MBI-GS), vicarious traumatization questionnaire, negative event questionnaire, Perceived Social Support Scale (PSSS) and coping style scale, was conducted one month after the release of the COVID-19 pandemic restrictions among resident training physicians of a tertiary first-class hospital in Zhejiang. The data was collected online from January 21 to February 9, 2023, through a mobile online questionnaire format^1^ (one widely used electronic survey platform in China). According to the official list of resident physicians in the hospital, we stented the QR code of the questionnaire to every resident physician one by one through an online work platform (“Ding-Ding”). The inclusion criteria were: resident physicians, working in a tertiary first-class hospital in Zhejiang, obtaining informed consent. The exclusion criteria were: any other general or specialty physicians, or medical students or other medical workers. The survey platform did not allow the participants to left blank in the question, which helped us exclude the result of those participants. And we had checked the data, ensuring all the results we got were most-likely logical.

The data was gathered using an online, self-administered, anonymous survey method. At the beginning of the questionnaire, the informed consent form was placed to ensure every participant should provide their informed consent and make sure they are suitable for this survey before completing the survey questions. If the resident physicians did not offer their consent, they could not answer the following questions to finish this survey. The goal of the study and the procedures for completing it were explained in detail to the participants. The main part of the questionnaires consisted of general personal information and measure scales. General personal information included: age, gender, year of residency, marital status, training type, and educational level.

This study has been approved by the ethics committee of the Second Affiliated Hospital of Zhejiang University School of Medicine (the ethical committee’s reference number: 20230216).

### Assessment of anxiety, depression, somatic symptoms, job burnout, and vicarious trauma

Anxiety was evaluated via GAD-7 (26) which is an effective tool for sifting anxiety and evaluating its degree in clinical practice and study. It’s a 7-item scale with a 4-point rating system. It’s calculated by assigning scores of 0, 1, 2, and 3, to the response categories of “not at all,” “several days,” “more than half the days,” and “nearly every day,” respectively, and adding together the scores for the seven questions. Total scores range from 0 to 21. In this sample, the scale demonstrated good internal consistency (Cronbach’s alpha = 0.9389) and the scale was used in the study by Chen et al. (27). In our study, participants with GAD-7 scores ≥10 indicated remarkable anxious symptoms.

The PHQ-9 (28) was employed to assess the level of depressive symptoms among resident physicians. It’s a 9-item scale developed for assessing depressive disorders in primary care populations. It’s calculated by assigning scores of 0, 1, 2, and 3, to the response categories of “not at all,” “several days,” “more than half the days,” and “nearly every day,” respectively, and adding together the scores for the nine questions. Total scores range from 0 to 27. In this sample, the scale demonstrated good internal consistency (Cronbach’s alpha = 0.9234) and the scale was used in the study by Chen et al. (27). In our study, participants with PHQ-9 scores≥10 were considered as having depression status.

The PHQ-15 (29) was employed to assess somatic symptoms. Each item is calculated by assigning scores of 0, 1, and 2, to the response categories of “not at all,” “a little,” and “often,” respectively, and adding together the scores for the 15 questions. Total scores range from 0 to 30. In this sample, the scale demonstrated good internal consistency (Cronbach’s alpha = 0.9081) and the scale was used in the study by Xuedong et al. (30). In our study, participants with PHQ-15 scores≥10 were considered as having somatic symptoms.

The Maslach Burnout Inventory-General Survey (MBI-GS) (31) was used to assess the job burnout of resident physicians. There are 22 items with a 7-point rating system. The items of the scale are divided into three dimensions including emotional exhaustion, personal accomplishment and depersonalization, the scores are calculated separately. Resident physicians select relevant items based on their own situation in the past month. In this sample, the scale demonstrated good internal consistency (Cronbach’s alpha = 0.8839) and the scale was used in the study by Gao et al. (32). In our study, participants defined the presence of job burnout symptoms by the median of the scale.

The vicarious trauma section of this study consisted of 29 questions, which were developed by the Department of Psychology and Behavioral Science of Zhejiang University specifically for medical personnel (33). Response options were “very mismatched,” “relatively mismatched,” “neutral,” “relatively matched,” and “very matched” scored as 0, 1, 2, 3 and 4. In this sample, the scale demonstrated good internal consistency (Cronbach’s alpha = 0.9679). In our study, participants defined the presence of vicarious trauma symptoms by the median of the scale.

### Assessment of the impact of negative events, social support, coping style

In this survey, we measured the impact of negative events, social support, coping style, vicarious trauma, and job burnout. To fit the clinical work environment discussed in this study, the scales we have chosen were as follows:

The negative event assessment was a series of 9 questions closely related to residents, namely, “relatives and friends are ill/seriously injured/dead,” “spouse is ill/seriously injured/dead,” “I am ill/seriously injured,” “financial difficulties,” “work and study pressure,” “conflict with colleagues or superiors,” “major changes in life rules (such as diet, sleep, and exercise),” “death of the patient in charge” and “other events.” Select “yes” or “no.” If “yes” was selected, further evaluation of the impact of the negative event was needed from 0 to 10.

The social support section used the Perceived Social Support Scale (PSSS), consisting of 12 questions (34). Response options were “strongly disagree,” “very disagree,” “slightly disagree,” “neutral,” “slightly agree,” “very agree,” and “strongly agree,” which were scored as 0, 1, 2, 3,4,5,6,7. The total score ranges from 12 to 84. A score between 12 and 36 indicates low support, 37–60 indicates medium support and 61–84 indicates high support (35). In this sample, the scale demonstrated good internal consistency (Cronbach’s alpha = 0.9278) and the scale was used in the study by Guo et al. (36).

The coping style section included 36 questions (37). Response options were “never taken,” “occasionally taken,” “sometimes taken,” and “often taken” scored as 0, 1, 2, 3. Determine whether the resident physicians were problem-centered or emotional coping styles based on this scale. In this sample, the scale demonstrated good internal consistency (Cronbach’s alpha = 0.8788).

Other information collected in the questionnaire included work status (whether you were at work after lifting the lockdown), service category (whether there was a temporary change of job position in the past month), workload (average clinical working hours per week in the past month), daily sleep duration (average sleep time on working days in the past month).

### Statistical analysis

Data analysis was conducted using STATA15.1. The Shapiro–Wilk test was used to examine the normality of continuous variables. Spearman correlation analysis was used to evaluate the correlation coefficients between various variables. According to the above scale norms, we have divided depression, anxiety, somatic symptoms, job burnout, and vicarious trauma scores into two types. The proportion of anxiety, depression, and somatic symptoms was calculated among resident physicians. To examine the differences between independent variables and the risk of depression, anxiety, somatic symptoms, job burnout, and vicarious trauma, Chi-square tests, and U-tests were used. A collinearity test was performed on the independent variable, and the variance expansion factor was calculated; we analyzed between independent variables and anxiety, depression, somatic symptoms, job burnout, and vicarious trauma in univariate logistic regression and multivariate logistic regression and calculated *OR* (odds ratio) and 95% *CI* (confidence interval). All tests were two-tailed, with a significance level of *p* < 0.05.

## Results

### Characteristics of the study participants

In this survey, 340 resident physicians completed all the required questionnaires. The demographic details are presented in Table 1. The mean age of the participants was 26.02 ± 2.90 years. The proportion of females (54.12%) was slightly higher than males (45.88%). Among the participants, different year of residency accounted for 41.76% (first), 33.53% (second), and 24.71% (third) respectively. 87.35% of them were unmarried. About half of the resident physicians were commissioned by other hospitals to receive training at our Hospital. 76.18% of them had a bachelor’s degree. During this period, 92.65% of resident physicians participated in the medical care for COVID-19 patients; 85.59% of resident physicians worked more than 40 h per week, 39.41% worked more than 49 h per week; 55.29% of resident physicians had less than 7 h of sleep per day. The results of the current study showed that there was a significant relationship between the year of residency and the prevalence of depression symptoms (*p* = 0.019), with the highest percentage of symptoms occurring in the third year of residency (39.29%). There were significant relationships between the age (*p* = 0.022), year of residency (*p* = 0.033), workload (*p* = 0.011) and the prevalence of anxiety symptoms, with the highest percentage of symptoms occurring in the third year of residency (30.95%) and workload>49 h (29.10%). There were significant relationships between the educational level (*p* = 0.037), daily sleep duration (*p* < 0.001), and the prevalence of somatic symptoms, with the highest percentage of symptoms occurring in the master (51.35%) and daily sleep duration<7 h (51.06%). There were significant relationships between the educational level (*p* = 0.014), work status (*p* = 0.014), daily sleep duration (*p* = 0.037) and the prevalence of job burnout, with the highest percentage of symptoms occurring in the undergraduate (55.60%), absent from work (76.00%), daily sleep duration<7 h (58.51%). There was a significant relationship between the gender and the prevalence of vicarious trauma (*p* = 0.015), with the higher percentage of symptoms occurring in man (58.33%).

**Table 1:** Demographic characteristics of participants (*N* = 340).

Variables	Category	*N* (%)	PHQ-9		GAD-7		PHQ-15		Job burnout		Vicarious trauma	
			χ^2^/z	*p*	χ^2^/z	*p*	χ^2^/z	*p*	χ^2^/z	*p*	χ^2^/z	*p*
Age (mean ± SD)	23–39 years	26.02 ± 2.90	−1.129	0.259	−2.287*	0.022	−0.533	0.594	−1.766	0.077	−0.561	0.574
Gender	Male	156 (45.88)	1.754	0.185	2.109	0.146	1.582	0.208	0.89	0.345	5.909*	0.015
	Female	184 (54.12)										
Year of residency	First year	142 (41.76)	7.901*	0.019	6.848*	0.033	4.351	0.114	2.856	0.24	2.699	0.259
	Second year	114 (33.53)										
	Third year	84 (24.71)										
Marital status	Never married	297 (87.35)	0.975	0.323	0.168	0.682	0.003	0.956	2.172	0.14	0.105	0.746
	Married	43 (12.65)										
Educational background	Undergraduate	259 (76.18)	0.326	0.85	1.322	0.516	6.611*	0.037	8.575*	0.014	2.145	0.342
	Master	37 (10.88)										
	Doctor	44 (12.94)										
Work status	On the job	315 (92.65)	0.004	0.951	0.013	0.91	0.024	0.877	6.049*	0.014	1.349	0.245
	Absent from work	25 (7.35)										
Service category	Temporary transformation	111 (32.65)	0.116	0.733	0.644	0.422	0.059	0.809	2.712	0.1	3.262	0.071
	Non-transformation	229 (67.35)										
Workload (hours/week)	<40 h	49 (14.41)	2.821	0.244	9.074*	0.011	4.606	0.100	4.276	0.118	4.381	0.112
	40-49 h	157 (46.18)										
	>49 h	134 (39.41)										
Daily sleep duration	<7 h	188 (55.29)	7.601*	0.022	4.371	0.112	16.018***	<0.001	6.570*	0.037	4.667	0.097
	7-8 h	144 (42.35)										
	>8 h	8 (2.35)										

*p < 0.05; ^**^p < 0.01; ^***^p < 0.001.

### Distributions of anxiety, depression, and somatic symptoms

The prevalence of anxiety (GAD-7 ≥ 10), depression (PHQ-9 ≥ 10), and somatic symptoms (PHQ-15 ≥ 10) in this study reached 20.88, 28.53, and 41.47%, respectively. Details are shown in Table 2.

**Table 2:** The prevalence of anxiety, depression and somatic symptoms.

Variables	Cut off score	Frequency	Percentages
GAD-7	<10	269	79.12
	≥10	71	20.88
PHQ-9	<10	243	71.47
	≥10	97	28.53
PHQ-15	<10	199	58.53
	≥10	141	41.47

### Related factors of anxiety, depression, somatic symptoms, job burnout, and vicarious trauma

Female resident physicians were more likely to experience somatic symptoms [adjusted *OR* = 2.36, 95% *CI*: 1.33–4.18] compared with males. The second-year resident physicians were less likely to report depression (adjusted *OR* = 0.41, 95% *CI*: 0.19–0.91) compared with the first-year resident physicians. Compared with resident physicians with a bachelor’s degree, resident physicians with a doctoral degree were less likely to experience somatic symptoms and job burnout (adjusted *OR* = 0.25, 95% *CI*: 0.09–0.67; adjusted *OR* = 0.31, 95% *CI*: 0.11–0.89). Resident physicians who were not on duty or had not been transferred were less likely to experience vicarious trauma symptoms (adjusted *OR* = 0.21, 95% *CI*: 0.06–0.73; adjusted *OR* = 0.35, 95% *CI*: 0.18–0.64). Resident physicians’ problem-focused coping styles were less prone to depression (adjusted *OR* = 0.92, 95% *CI*: 0.88–0.96), anxiety (adjusted *OR* = 0.94, 95% *CI*: 0.90–0.98), somatic symptoms (adjusted *OR* = 0.93, 95% *CI*: 0.89–0.97), job burnout (adjusted *OR* = 0.91, 95% *CI*: 0.87–0.96), and vicarious trauma (adjusted *OR* = 0.94, 95% *CI*: 0.90–0.98). Resident physicians’ emotion-focused coping styles and who experienced negative life events were more prone to anxiety (adjusted *OR* = 1.13, 95% *CI*: 1.08–1.17; adjusted *OR* = 1.05, 95% *CI*: 1.03–1.08), depression (adjusted *OR* = 1.11, 95% *CI*: 1.07–1.16; adjusted *OR* = 1.05, 95% *CI*: 1.03–1.07), somatic symptoms (adjusted *OR* = 1.10, 95% *CI*: 1.06–1.14; adjusted *OR* = 1.06, 95% *CI*: 1.04–1.08), job burnout (adjusted *OR* = 1.16, 95% *CI*: 1.11–1.20; adjusted *OR* = 1.03, 95% *CI*: 1.01–1.06), and vicarious trauma (adjusted *OR* = 1.13, 95% *CI*: 1.09–1.17; adjusted *OR* = 1.05, 95% *CI*: 1.02–1.07). Details are shown in Table 3.

**Table 3:** Multivariate logistic regression analysis on resident physicians of factors influencing psychological status.

Variables		PHQ-9		GAD-7		PHQ-15		Job burnout		Vicarious trauma	
		Adjusted *OR* (95%*CI*)	*p*	Adjusted *OR* (95%*CI*)	*p*	Adjusted *OR* (95%*CI*)	*p*	Adjusted *OR* (95%*CI*)	*p*	Adjusted *OR* (95%*CI*)	*p*
Age		1.09 (0.95–1.25)	0.218	1.13 (0.98–1.31)	0.093	1.10 (0.96–1.26)	0.168	1.02 (0.89–1.18)	0.782	1.10 (0.97–1.24)	0.148
Gender		0.87 (0.48–1.56)	0.640	0.91 (0.48–1.72)	0.772	2.36 (1.33–4.18)**	0.003	0.85 (0.47–1.53)	0.581	0.62 (0.36–1.06)	0.080
Year of residency	First year	Reference		Reference		Reference		Reference		Reference	
	Second year	0.41 (0.19–0.91)*	0.028	0.82 (0.35–1.92)	0.646	1.12 (0.57–2.19)	0.749	0.70 (0.34–1.45)	0.336	0.72 (0.38–1.39)	0.332
	Third year	1.17 (0.57–2.41)	0.666	1.71 (0.78–3.75)	0.178	1.28 (0.63–2.60)	0.503	1.08 (0.50–2.31)	0.853	1.11 (0.56–2.21)	0.755
Marital status		0.80 (0.30–2.11)	0.654	1.61 (0.57–4.55)	0.369	1.27 (0.47–3.45)	0.637	1.35 (0.47–3.91)	0.576	1.14 (0.44–2.94)	0.788
Educational background	Undergraduate	Reference		Reference		Reference		Reference		Reference	
	Master	0.99 (0.38–2.59)	0.977	0.98 (0.35–2.79)	0.976	1.04 (0.42–2.54)	0.935	0.82 (0.31–2.20)	0.691	1.14 (0.47–2.78)	0.776
	Doctor	0.71 (0.27–1.89)	0.490	1.18 (0.43–3.23)	0.752	0.25 (0.09–0.67)**	0.006	0.31 (0.11–0.89)*	0.028	0.50 (0.20–1.23)	0.131
Work status		0.47 (0.12–1.83)	0.274	0.78 (0.19–3.28)	0.738	0.52 (0.15–1.84)	0.312	1.64 (0.40–6.72)	0.490	0.21 (0.06–0.73)*	0.013
Service category		0.69 (0.36–1.36)	0.286	0.73 (0.35–1.52)	0.405	0.77 (0.41–1.43)	0.402	0.54 (0.28–1.05)	0.071	0.35 (0.18–0.64)**	0.001
Workload (hours/week)	<40 h	Reference		Reference		Reference		Reference		Reference	
	40-49 h	0.84 (0.32–2.23)	0.722	0.82 (0.27–2.47)	0.722	0.88 (0.35–2.22)	0.779	0.49 (0.17–1.37)	0.172	0.71 (0.29–1.72)	0.443
	>49 h	0.96 (0.35–2.62)	0.933	1.65 (0.54–5.01)	0.381	1.25 (0.47–3.33)	0.651	0.44 (0.15–1.30)	0.138	1.12 (0.44–2.83)	0.818
Daily sleep duration	<7 h	Reference		Reference		Reference		Reference		Reference	
	7-8 h	0.73 (0.39–1.34)	0.308	1.09 (0.56–2.14)	0.797	0.56 (0.32–1.00)	0.050	0.61 (0.33–1.12)	0.113	0.93 (0.53–1.62)	0.795
	>8 h	2.10 (0.29–15.17)	0.464	1.57 (0.15–16.59)	0.709	0.64 (0.10–4.19)	0.636	0.17 (0.02–1.69)	0.130	3.83 (0.73–20.08)	0.112
Understanding social support		0.99 (0.96–1.01)	0.291	1.00 (0.97–1.02)	0.720	0.98 (0.96–1.01)	0.230	0.94 (0.91–0.97)***	<0.001	1.01 (0.98–1.03)	0.694
Response to the problem		0.92 (0.88–0.96)***	<0.001	0.94 (0.90–0.98)**	0.008	0.93 (0.89–0.97)**	0.001	0.91 (0.87–0.96)***	<0.001	0.94 (0.90–0.98)**	0.002
Response to the emotion		1.11 (1.07–1.16)***	<0.001	1.13 (1.08–1.17)***	<0.001	1.10 (1.06–1.14)***	<0.001	1.16 (1.11–1.20)***	<0.001	1.13 (1.09–1.17)***	<0.001
Negative events		1.05 (1.03–1.07)***	<0.001	1.05 (1.03–1.08)***	<0.001	1.06 (1.04–1.08)***	<0.001	1.03 (1.01–1.06)**	0.010	1.05 (1.02–1.07)***	<0.001

*p < 0.05; ^**^p < 0.01; ^***^p < 0.001.

## Discussion

This online survey investigates the psychological status of resident physicians in a tertiary hospital in the early stages of lifting the COVID-19 lockdown in China. The uniqueness of this study lay in the series of questionnaire surveys we conducted on a specific population in a special background, in addition to evaluating anxiety and depression, it also included relevant assessments of somatic symptoms, job burnout, and vicarious trauma among resident physicians. The main findings of this study are as follows. Firstly, in the early stages of lifting the COVID-19 lockdown in China, the prevalence of anxiety, depression, and somatic symptoms among resident physicians in the standardized training stage was high. An important finding was that 41.47% of resident physicians had significant somatic symptoms. We also found that there was a correlation between sleep time and somatic symptoms, and the shorter the sleep time, the more likely to experience somatic symptoms such as fatigue. Secondly, the study also found a correlation between certain psychological problems and age, gender, year of residency, and educational level. Thirdly, resident physicians who were not on duty or had not been transferred were less likely to experience vicarious trauma symptoms. Fourthly, adequate social support was less likely to lead to job burnout. Lastly, resident physicians’ problem-focused coping styles were less prone to depression, anxiety, somatic symptoms, job burnout, and vicarious trauma; Resident physicians’ emotion-focused coping styles and experienced negative life events were more prone to anxiety, depression, somatic symptoms, job burnout, and vicarious trauma. Then, we will discuss our findings separately.

After the outbreak of COVID-19, China has been prevented and controlled for nearly three years, and there has been no large-scale spread. On December 26, 2022, the National Health Commission issued a notice: With the approval of the State Council, as of January 8, 2023, the measures for the prevention and control of Class A infectious diseases stipulated in the Law of the People’s Republic of China on the Prevention and Control of Infectious Diseases against COVID-19 would be lifted. COVID-19 outburst in the short term. Almost everyone was infected with COVID-19. All wards were receiving patients with COVID-19. Medical workers face multiple physical and psychological challenges. In our study, we found that in the early stages of lifting the COVID-19 lockdown in China, the prevalence of anxiety, depression, and somatic symptoms among resident physicians in the standardized training stage were 20.88, 28.53, and 41.47%. Previous studies have also shown that resident physicians had high levels of depression and emotional problems (8, 9, 38). The results of one system review showed that 20.9–43.2% of resident physicians had depression and depressive symptoms (9). An important finding was that about half of resident physicians had more somatic symptoms. We analyzed that this phenomenon might be related to a sharp increase in workload and a decrease in sleep time. Correlation analysis in our study indeed found a remarkable negative association between daily sleep time and somatic symptoms. Research has found that in addition to working hours (10), the sleep time (39) and poor sleep quality (40) of resident physicians were also related to fatigue. Fortier’s study has shown a potential cross-sectional relationship between insomnia and fatigue (41). Many studies confirmed that sleep time was an important predictor of fatigue the next day (42, 43). Insufficient sleep (≤ 6 h per night) increases the risk of depression among resident physicians and might lead to higher medical error rates (39, 44, 45).

In our study, we found a positive association between age and anxiety. Medical students in the higher (>22 years) age group in Nepal suffered from elevated levels of anxiety and/or depression during COVID-19 (46). The age of resident physicians in our study was similar to that of medical students in Nepal and similar conclusions were found. The reason for similar conclusions might be related to the similarity of research background and population. We also found gender and grade differences in certain assessment content. For example, compared to males, female resident physicians were more likely to experience somatic symptoms. In the past, many studies focused on the somatic symptoms of peri-menopausal (47) and older females (48), and compared to males, the prevalence of somatic symptoms in females during this period were higher. Although this conclusion was similar to ours and had some reference value, comparability decreased due to other factors such as age and occupation. However, there was also a study that suggested that females were seeking medical help more frequently due to somatic symptoms (49). We also found similar conclusions regarding the specific female population in our study, which was also a part of the female population. Compared with first-year resident physicians, resident physicians in the second year of training stage were less likely to report depression, which was related to the fact that second-year resident physicians had a relatively easy year, unlike the first-year resident physicians who just arrived in the new environment and was in the adaptation stage, also unlike the third-year resident physicians who at the last year of standardized training and faced more challenges and tests, bore more work responsibilities and also faced more pressure in terms of assessment. Moreover, we found that compared with resident physicians with a bachelor’s degree, resident physicians with a doctoral degree were less likely to experience somatic symptoms and job burnout; More research on PhD students showed that PhD students demonstrated a remarkably higher level of emotional problems, somatic symptoms and sleep issues compared to persons who had not received further education after obtaining a master’s degree (50). A previous study about academic medicine faculty found that teachers with master’s and doctoral degrees had a higher risk of depression and anxiety (51). Our research conclusions seemed to be inconsistent with many previous research conclusions. One possible reason was that our research subjects were resident physicians who had already worked and were not students on campus. Additionally, we concluded that resident physicians were less likely to experience somatic symptoms and job burnout. Previous studies have focused more on anxiety and depression symptoms. Further research is needed to analyze the specific reasons in the future.

Our study found that resident physicians who were not on duty or had not been transferred were less likely to experience vicarious trauma symptoms; some resident physicians were not on duty due to policy reasons or COVID-19 infection, they seldom contacted or managed COVID-19 patients, so they were less impacted and less likely to have vicarious trauma symptoms. Many resident physicians have been transferred from their original posts to emergency, infection department, intensive care unit, and other departments due to work needs, and were more likely to have vicarious trauma symptoms when facing COVID-19 patients directly.

Good social support was less likely to lead to job burnout; Similar to a previous study that found that organizational support reduced burnout (52). Cardozo et al. also found that social support and healthy coping strategies were protective factors for depression, anxiety, and burnout (53). Extremely amount of work, document burden, declined regulation over workload, difficulty integrating into work and life, and loss of meaning in work were elements related to burnout (14). A multicenter prospective study on the pressure, anxiety, and job burnout of medical workers during COVID-19 in Singapore found that long working hours were closely related to anxiety and job burnout (12). A reasonable response to external stress and sufficient social support might reduce the occurrence of emotional problems and job burnout.

The study suggested that resident physicians’ problem-focused coping styles were less prone to depression, somatic symptoms, job burnout, and vicarious trauma; while resident physicians were more prone to feel anxiety, depression, somatic symptoms, job burnout, and vicarious trauma through emotion-focused coping styles. The found were similar to many researches. Cruz et al. found that problem-focused coping was negatively associated with depressive symptoms and impairments of social. And there was a remarkable positive association between emotion-focused coping and depressive symptoms (54). When facing negative stress events, positive coping styles were protective factors for anxiety and depression, while avoidance coping styles were risk factors for anxiety and depression (55, 56). Yan et al. also found that people who used positive coping measures felt fewer symptoms of depression, obsessive-compulsive anxiety, and neurasthenia under stress, while negative coping measures aggravated mood distress (57). Our study found a correlation between job burnout and coping styles. A major factor that might influence job burnout is a person’s coping style, which is the cognitive and behavioral efforts they make to cope with stress (54). Coping has two primary roles: adjusting unstable emotions and resolving emotional distress through cognitive and behavioral changes (58–60). The use of adaptive coping strategies has a positive impact on the body and mental health, stress management, and overall performance of medical staff (61). We can improve the psychological health status of resident physicians by educating them on reasonable coping strategies.

Negative life events were adverse factors for anxiety, depression, somatic symptoms, job burnout, and vicarious trauma. The more negative events, the easier it was to experience anxiety, depression, somatic discomfort, job burnout, and vicarious trauma. One study related to negative life events and mental health in adolescents, found the more frequent and intensive negative life events that adolescents experienced, the more likely they were to experience symptoms of depression and anxiety (62). Our study also collected and analyzed the negative events experienced by resident physicians within one month of the release of the COVID-19 pandemic restrictions. In the early stages of lifting the COVID-19 lockdown, everyone more or less experienced negative events related to the disease, and psychological health is hypersensitive to trauma incidents and their social and commercial outcomes (60). The pandemic was a crash process that endangered individual’s life and survival, with many traumatic effects on everyone’s physical and psychological well-being (59). Compared to the general population, resident physicians needed to undertake more work tasks and faced greater risks of infection, even had to work with diseases, in such difficult situation. The negative events experienced during this period were more frequent than usual, which might lead to a higher prevalence of anxiety, depression, somatic symptoms, job burnout, and vicarious trauma.

Restricted by special periods and populations, some limitations should be considered when explaining the findings of the present study. Firstly, all information was based on the self-report of the resident physicians. Secondly, all resident physicians’ data comes from only one large hospital, due to the impact of special period, the number of collected samples was relatively small, which may not represent all resident physicians in the standardized training stage. A larger multicenter sample size was needed to increase the representativeness of the data. Thirdly, this was a descriptive study, and confirming causal relationships was relatively difficult. Further longitudinal research should be utilized to address this relationship. Nevertheless, our study has added a survey of favorable factors related to psychological health status, which could provide a basis for developing relevant psychological intervention measures. Cognitive behavioral therapy (CBT) and crisis intervention have been considered helpful intervention strategies for managing the psychological health results of medical staff (63). In addition, protective and risk factors for anxiety, depression, somatic symptoms, job burnout, and vicarious trauma identified among resident physicians provided valuable information for developing relevant psychological intervention measures to improve the psychological health of relevant groups after the outbreak of infectious diseases. Further longitudinal researches also need to be confirmed.

## Conclusion

This study found that resident physicians had a high prevalence of anxiety, depression, and somatic symptoms during the release of COVID-19 pandemic restrictions. Internal and external factors of residents were correlated with the occurrence of anxiety, depression, somatic symptoms, job burnout, and vicarious trauma. Risk and protective factors related to the psychological health of residents have been proposed. These findings suggested that interventions aimed at reducing working hours and workload, ensuring sufficient sleep time, promoting problem-focused coping strategies, strengthening regular communication and mental health education or intervention, might help improve the psychological health status of resident physicians and their ability to cope with complex tasks.

## Data availability statement

The raw data supporting the conclusions of this article will be made available by the authors, without undue reservation.

## Ethics statement

The studies involving humans were approved by the ethics committee of the Second Affiliated Hospital of Zhejiang University School of Medicine. The studies were conducted in accordance with the local legislation and institutional requirements. The participants provided their written informed consent to participate in this study.

## Author contributions

QZ: Conceptualization, Writing – original draft, Writing – review & editing. RP: Data curation, Formal analysis, Writing – original draft, Writing – review & editing. QP: Data curation, Formal analysis, Writing – review & editing. YQ: Formal analysis, Writing – review & editing. XZ: Conceptualization, Writing – review & editing. QC: Conceptualization, Methodology, Project administration, Resources, Writing – review & editing.

## Footnotes

^1^ http://www.wjx.cn
